# Analysis of the Testimony on the Trial of Alvin Coswell, for Murder, and of the Subsequent Proof Which Led to the Commutation of His Punishment

**Published:** 1846-06-01

**Authors:** 


*Analysis of the Testimony on the Trial of Alvin Coswell, for Murder, and of the subsequent Proof which led to the Commutation of his Punishment. Communicated by* Drs. T. R. Beck and Brigham.

This is a highly interesting and valuable article, involving one of the nicest and most momentous questions in forensic medicine, viz:—the existence of moral responsibility for the commission of homicide. The article is particularly worthy of examination, as it contains the facts of a case in which a jury returned a verdict of wilful murder, and the sentence of death was pronounced by the presiding judge, but in which a commutation was based upon the opinions of two medical men of distinguished attainments in the branch of medical investigations immediately concerned in the questions at issue.

The article, being itself an analysis, does not admit of condensation, so that we can only commend it to the perusal of our readers.

It is honorable to the feelings and intelligence of Gov. Bouck that he consented to submit the question upon which executive justice was demanded, to a proper and competent tribunal. It redounds to his honor, because this principle of action is not always recognised in such cases. Compare with this instance that of judicial murder noticed in the 10th No. of our first volume, which occurred in a neighboring state. Humanity has abundant cause for rejoicing in the progress of medical science if we were to look no farther than the details of such a case as is communicated by Drs. Beck and Brigham.

[*Notice of the Proceedings of the State Medical Society we reserve for a future number.*]

